# Drug-eluting beads transarterial chemoembolization vs conventional transarterial chemoembolization in the treatment of hepatocellular carcinoma in adult patients: a systematic review and update meta-analysis of observational studies

**DOI:** 10.3389/fonc.2024.1526268

**Published:** 2025-02-12

**Authors:** Tatiana Chernyshenko, Roman Polkin, Ekaterina Dvoinikova, Valeriy Shepelev, Roman Goncharuk

**Affiliations:** ^1^ Department of Surgery, Far Eastern Federal University, Vladivostok, Russia; ^2^ Medical Center, Far Eastern Federal University, Vladivostok, Russia; ^3^ Department of Neurosurgery, 1477th Naval Clinical Hospital, Vladivostok, Russia

**Keywords:** transarterial chemoembolization, drug-eluting beads, hepatocellular carcinoma, systematic review, meta-analysis

## Abstract

**Study design:**

Systematic review and update meta-analysis.

**Purpose:**

The present systematic review and meta-analysis were conducted to compare the efficacy and safety of the two approaches for HCC in adult patients (DEB-TACE vs cTACE).

**Overview of literature:**

The TACE procedure is indicated for the treatment of HCC with intermediate (BCLC B) and early (BCLC A). Conflicting data obtained from earlier meta-analyses comparing DEB-TACE with cTACE prompted the updated meta-analysis.

**Methods:**

The study included adult patients over the age of 18 with HCC. MEDLINE conducted a literature search using Pubmed and Google Scholar up to May 2024. The following parameters were evaluated: the effectiveness of the tumor response to treatment according to the mRECIST criteria (CR, PR, SD, PD), overall survival, progression-free survival, and complication rate. 32 retro- and prospective studies were analyzed.

**Results:**

The study included 4,367 patients. The radiological response of the tumor in all four CR, PR, SD, and PD parameters in the DEB-TACE group showed the best response. The overall survival rate during the DEB-TACE procedure was higher by 3.54 months (p <0.00001), and progression-free survival (PFS) by 3.07 months (p <0.0001), respectively. The incidence of complications was comparable in both groups.

**Conclusions:**

The results of the meta-analysis revealed clinically significant advantages of DEB-TACE in comparison with cTACE. Being comparable in terms of the frequency of complications, DEB-TACE demonstrated the best result in the radiological response of the tumor to the therapy, in terms of overall survival and progression-free survival.

## 1 Introduction

Liver cancer is the sixth most common in the world and ranks third as the cause of death from malignant neoplasms (MNP) (1).

Hepatocellular carcinoma (HCC) and cholangiocarcinoma are the two most common primary liver MNPs. Hepatocellular carcinoma develops from hepatocytes, and cholangiocarcinoma develops from bile duct cells (2).

HCC accounts for 75-85% of all primary liver MNPs, leading to the fourth most common cause of cancer-related death in the world (3).

Existing methods of treating HCC, such as surgical resection, transplantation, systemic drug therapy, and stereotactic irradiation, are complemented by the use of minimally invasive methods. One of these options is transarterial chemoembolization (TACE), which consists of the administration of chemotherapeutic drugs directly into the artery feeding the tumor under conditions of digital subtraction angiography.

TACE is performed in the treatment of HCC with intermediate (BCLC B) and early (BCLC A) stages according to the BCLC classification (4). Classical transarterial chemoembolization (cTACE) and transarterial chemoembolization using drug-eluting beads (DEB-TACE) are the two main options for locoregional treatment (5).

cTACE is a procedure that involves the sequential delivery of a chemotherapeutic drug and lipidol into the vessels feeding the tumor, followed by an embolic agent (6).

DEB-TACE is another type of TACE that contains beads saturated with the drug. The use of this technique makes it possible to increase the concentration of the drug in the tumor and reduce its systemic concentrations compared to cTACE (4). However, the disadvantages of DEB-TACE are the constant occlusion of the artery feeding the tumor due to non-degradable beads and a limited choice of therapeutic agents for loading (7, 8).

At the moment, the algorithms for selecting a chemotherapeutic drug and the method of its delivery based on the morphological subtype of the tumor and the stage of the disease remain the subject of active discussions. Conflicting data obtained from previously conducted meta-analyses (16–19) comparing DEB-TACE with cTACE led to the publication of new clinical studies, which prompted the implementation of an updated meta-analysis.

## 2 Materials and methods

This review was conducted in accordance with the recommendations of The Preferred Reporting Items for Systematic Reviews and Meta-Analyses (PRISMA) (9) and Assessment of Multiple Systematic Reviews AMSTAR. A systematic search was conducted via MEDLINE, PubMed, and Google Scholar. A highly sensitive search strategy using keywords was used for the search: hepatocellular carcinoma AND transarterial chemoembolization, hepatocellular carcinoma, AND chemoembolization, drug-eluting beads AND hepatocellular carcinoma. Irrelevant studies were excluded and duplicates were deleted. Only original articles from 2010 to 2024 were selected. Additional links were found by manually searching the literature lists of relevant studies, conference abstracts, and registered clinical trials. The search was limited to publications in English.

### 2.1 Selection criteria

All articles were selected using previously specified keywords. The data were independently selected by two authors (TC, RP), who checked all relevant titles and abstracts of publications to exclude irrelevant ones. The researchers independently evaluated the complete reports, after which each selected article was independently evaluated by the entire author’s team using PICOS (Population, Intervention, Comparison, Outcome, Study Design) (10) inclusion and exclusion criteria (**Table 1**).

**Table 1:** PICOS. Inclusion and exclusion criteria.

PICOS	Inclusion criteria	Exclusion criteria
Population	Patients over 18 years of age, hepatocellular cancer	Patients under 18 years of age, metastatic cancer, cholangiocellular cancer
Intervention	Transarterial chemoembolization using drug-eluting beads (DEB-TACE)	Transarterial chemoembolization with degradable beads (DSM-DEB)
Comparison	Transarterial Chemoembolization (cTACE)	Transarterial radioembolization
Outcome	Evaluation of the effectiveness of the tumor response to treatment according to the mRECIST criteria (CR, PR, SD, PD) or overall survival, progression-free survival,	Incomplete information on one of the criteria
Study design	Randomized control studies, non-randomized prospective and retrospective observational studies	Case reports, systematic reviews, meta-analyses, preclinical studies
Publications	Full-text publications in English	Publications in other languages, unpublished research, protocols, conference and presentation materials, abstracts, surgical videos

### 2.2 Data extraction and quality assessment

The two above-mentioned authors independently extracted data using standardized forms. From publications that meet the inclusion criteria, information on the year, study design, type of emboli, intervention, comparative control, overall survival, mean and standard deviations (SD) or confidence interval (CI), as well as sample sizes were obtained. Modified scales were used to assess the methodological quality of research: Newcastle-Ottawa, NIH quality assessment tool for case series studies, and Cochrane Risk of Bias (ROB) 2.0 tool (11).

### 2.3 Evaluation of outcomes

The study primarily analyzed the following parameters: (1) median overall survival, (2) progression-free survival, (3) radiological response to treatment, according to the recommendations of the “Criteria for Evaluating Response in Solid Tumors” (RECIST) (12), the frequency of complications during hospitalization.

### 2.4 Statistical analysis

To analyze the data, we used the Review Manager ver. 5.4 (The Nordic Cochrane Center, The Cochrane Collaboration, Copenhagen, Denmark). Risk ratio (RR), odds ratio (OR), and 95% confidence interval (CI) were calculated for dichotomous variables; standardized mean differences (SMD) and their 95% CI were used for continuous variables. The degree of heterogeneity was estimated using the coefficient I2. The fixed effects model was used for the absence of heterogeneity, and the random effects model was used if I2 was greater than 40%. A funnel-shaped graph was constructed and an Egger’s test was performed to assess the systematic error of the publication. A value of p <0.05 was used to indicate statistical significance. The standard deviations were calculated using the Cochrane Handbook for Systematic Reviews of Interventions (13).

## 3 Results

### 3.1 Systematic search results

**Figure 1:**
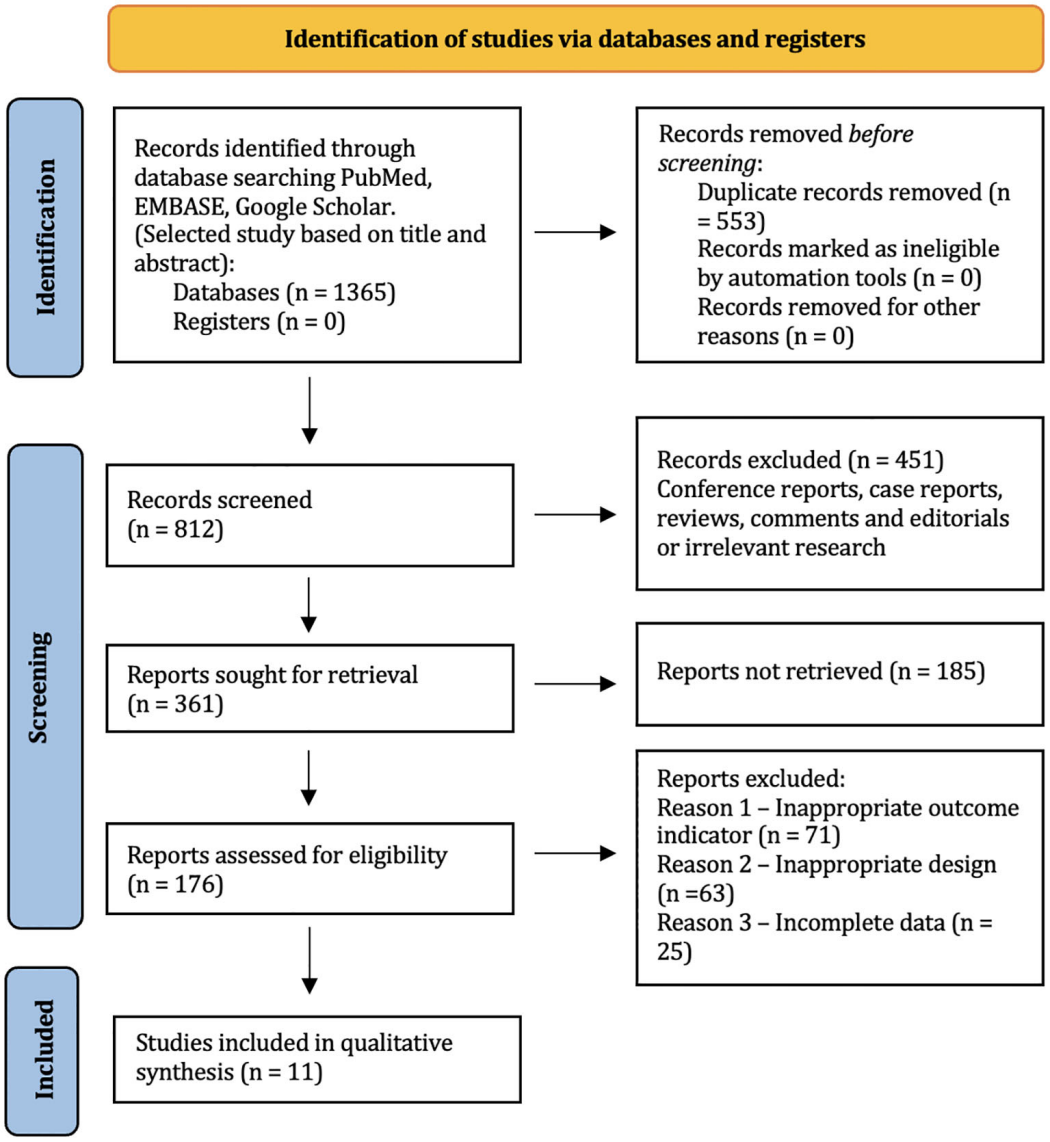
Flow diagram of the studies included in the systematic review according to PRISMA.

**Figure 1** shows a brief description of the research selection process. In total, 1,365 articles were found in the databases of MEDLINE via PubMed, and Google Scholar. A total of 1,189 studies were excluded because they were duplicates, irrelevant studies, case reports, and reviews. A total of 176 potential articles were received for further full-text evaluation. Of these, 157 articles were excluded for non-compliance with the inclusion criteria. The final synthesis included 32 studies. 11 of them were added as a result of an updated systematic search. **Table 2** summarizes the main characteristics of the included studies

**Table 2:** General characteristics of the studies included in the systematic review and meta-analysis.

No.	Title	Median follow up (mo)	Number of patients	Research design	Transarterial chemoembolization agent, DEB-TACE group	Transarterial chemoembolization agent, cTACE group	Main results	Newcastle-Ottawa Scale	CASP tool	ROB
1.	Arabi, M. et al. (24) (2015)	2.8	DEB-TACE=51 cTACE=25	Retrospective Case series	Doxorubicin 75 mg DC beads (Biocompatibles, Surrey, UK) 100–300 and 300–500 µm	Cisplatin 50–100 mg +Lipidol +Polyvinyl alcohol particles (Contour^®^ PVA Embolization Particles, Boston Scientific, Natick, MA, USA) 355–500 µm	The OS median was in the DEB-TACE group for 61 days and in the c-TACE group for 86 days. The indicators of CR, DC and PD were 11%, 53% and 47%, respectively, in the DEB-TACE group and 4%, 64% and 36% in the c-TACE group.	N/a	Fair	
2.	Bargellini I. et al. (25) (2021)	33.8	DEB-TACE=101 cTACE=101	Retrospective cohort study	Doxorubicin 50 mg or 75 mg DC- Beads (Biocompatibles UK Ltd; now a Boston Scientific Company) 100–300 μm.	Doxorubicin 50 mg or 75 mg odized oil (Lipiodol; Guerbet) + Gelatin sponge particles	cTASE showed a high incidence of side effects (p = 0.03). PFS and OS were comparable. The complete response (CR) was significantly (p = 0.009) better in the DEB-TACE group compared to cTACE.	***/*/***	N/a	
3.	Cai L. et al. (19) (2022)	12.4	DEB-TACE=20 cTACE=24	Retrospective cohort study	Epirubicin 60–80 mg CalliSpheres (Jiangsu Hengrui Medicine Co., Ltd., Jiangsu Province, China) 100–300 or 300–500 μm	Epirubicin 60–80 mg +Ethiodized poppyseed oil (EPO) (Jiangsu Hengrui Medicine Co., Ltd., Jiangsu Province, China) +PVA particles (CookMedical LLC, Bloomington, USA)	ORR was better in the DEB-TACE group compared to the cTACE group (P = 0.012). The PFS in the DEB-TACE group was 12.2 months (95% CI: 9.9–14.6) compared to 7.8 months (95% CI: 5.6–10.0) (P = 0.037). OS in the DEB-TACE group was 20.0 months (95% CI: 18.1–21.9) compared to 18.6 months (95% CI: 15.4–21.8) (P = .341).	**/**	N/a	
4.	Chiu S. et al. (26) (2020)	12.0	DEB-TACE=42 cTACE=19	Retrospective Case series	Doxorubicin 100 mg HepaSpheres (Merit Medical, South Jordan, Utah, USA) 30–60 μm	Doxorubicin 100 mg Lipiodol^®^ (Guerbet, France) + Gelfoam sponges	DEB-TACE showed better efficiency than cTACE in terms of OR (p=0.005), TTP (p=0.002) and OS (p=0.045). There was no significant difference in the occurrence of complications (p=0.72)	N/a	Good	
5.	Dhanasekaran R. et al. (27) (2010)	N/a	DEB-TACE=45 cTACE=26	Retrospective Case series	Doxorubicin 75 mg LC beads (Biocompatibles, Farnham, Surrey, UK) 300–500 and 500– 700mm	Doxorubicin 50 mg cisplatin 100 mg mitomycin 10mg + lipiodol (Ethiodol; Savage Laboratories, Melville, NY) + polyvinyl alcohol (PVA) particles.	The average OS time with DEB and cTACE was 610 days (351-868) and 284 days (4-563) P =0.03.	N/a	Good	
6.	Duan X. et al. (28) (2022)	6.1	DEB-TACE=31 cTACE=40	Retrospective cohort study	Pirarubicin 60–80 mg CalliSpheres (Jiangsu Hengrui Medicine Co., Ltd., Jiangsu Province, China) 300–500 mm	Pirarubicin 20 mg +Ethiodized poppyseed oil injection (EPO) (Jiangsu Hengrui Medicine Co., Ltd., China) + Gelatin sponge particles (Hangzhou Aili Pharmaceutical Technology Co., Ltd., China) 350–560 mm	DEB-TACE showed a higher ORR (60.0% vs. 29.7%, p <0.05). 3.3 months PFS (95% CI: 2.8–3.7) vs. 2.1 months (95% CI: 1.7–2.5), as well as OS 7.8 months (95% CI: 4.6–11.0) vs. 5.7 months (95% CI: 5.0–6.3) were better in the DEB-TACE group compared to the cTACE group (p <0.01).	**/**/***	N/a	
7.	Facciorusso A. et al. (29) (2016)	42	DEB-TACE=145 cTACE=104	Retrospective cohort study	Doxorubicin 50 mg DC beads^®^ (Biocompatibles, Surrey, UK) 100–300μm	Doxorubicin 50 mg Lipiodol + Gelfoam powder (Pfizer Inc., New York, NY, USA) 40 to 60 μm	PFS in the DEB-TACE group was 17 months (95% CL: 14-21) versus 11 months (95% CL: 9-12), (P<0.001). OS was 39 months (95% CL: 32-47) in cTACE and 32 months (95% CL: 24-39) in DEB-TACE.	***/*/***	N/a	
8.	Fan W. et al. (30) (2021)	11.4	DEB-TACE= 57 cTACE= 62	Retrospective Case series	Doxorubicin 75 mg DC Beads (Biocompatibles) 100–300 or 300–500 μm	Doxorubicin 50 mg +Lipiodol (Guerbet) +300–500-μm +trisacryl gelatin microspheres (Embosphere particles; Biosphere Medical)	The complication rate (45.6% vs. 79.0%, p <0.001) was significantly lower in the DEB-TACE group than in the cTACE group. The DEB-TACE group had better ORR, (70.2% vs. 50.0%). The OS and TTP median were longer in the DEB-TACE group (11.4 vs. 9.1 months, p <0.001; 6.9 vs. 5.2 months, p = 0.045).	N/a	Fair	
9.	Golfieri, R. et al. (31) (2014)	24.0	DEB-TACE= 89 cTACE= 88	RCT	Doxorubicin 50 mg DC-Beads (Biocompatibles, Farnham, Surrey, UK) 100–300 mm	Pirubicin 50 mg +iodized oil (Lipiodol; Guerbet, Milan, Italy) +gelatin sponge particles (Gelita-Spon; Gelita Medical, Amsterdam, The Netherlands)	The TTP median was 9 months in both groups. The frequency of adverse events was the same in both groups. Survival rates for 1 and 2 years were 86.2% and 56.8% after DEB-TACE and 83.5% and 55.4% after cTACE.	N/a	N/a	Low
10.	Hui Li et al. (32) (2019)	15.0	DEB-TACE= 42 cTACE= 39	Retrospective cohort study	Pirarubicin CalliSpheres (Jiangsu Hengrui Medicine Co., Ltd., Jiangsu Province, China) 300 to 500mm	Pirarubicin ethiodized poppyseed oil (EPO) (Jiangsu Hengrui Medicine Co., Ltd., Jiangsu Province, China) + Polyvinyl Alcohol (PVA) particles (Cook Medical LLC, Bloomington, IN)	CR (P=.167) was the same between the 2 groups. Patients in the DEB-TACE group had better PFS (P = .028) and OS (P = .037) compared to the cTACE group. The frequency of pain (P=.327), fever (P=.171) and nausea/vomiting (P=.400) during hospitalization were the same between the 2 groups.	**/**/***	N/a	
11.	Kloeckner, R. et al. (33) (2015)	18.0	DEB-TACE= 76 cTACE= 174	Retrospective Case series	150 mg doxorubicin DcBeads^®^ [Biocompatibles, Farnham, UK] 500–700 μm, 300-500 μm, and 100-300 μm;	10 mg Mitomycin iodized oil [Lipiodol Ultra-Fluide^®^; Guerbet Laboratories, Aulnay-Sous-Bois, France] or polyvinyl alcohol particles	OS was 409 days (95% CI: 321-488 days) in the cTACE group and 369 days (95% CI: 310-589 days) in the DEB-TACE group (p = 0.76)	N/a	Good	
12.	Kucukay F. et al. (34) (2015)	N/a	DEB-TACE= 53 cTACE= 73	Retrospective Case series	doxorubicin HepaSphere particles (Biosphere Medical, Roissy-en-France, France) 30–60-μm	doxorubicin Embosphere particles and Gelfoam (Pharmacia & Upjohn, Kalamazoo, Michigan)	OS was 37.4 (30.9–43.8) in the DEB-TACE group and 39.0(31.4–46.6) in cTACE	N/a	Fair	
13.	Lammer J. et al. (35) (2010)	6	DEB-TACE= 89 cTACE= 100	RCT	150 mg doxorubicin DC Bead (Biocompatibles UK Ltd.) 300–500 lm 500–700 lm)	50-150 mg doxorubicin Lipiodol (iodinated poppy seed oil; Guerbet, France) Gelfoam particles, Embosphere, Contour SE, Bead Block, PVA particles	CR, ORR, and DCR were better in the DEB-TACE group compared to cTACE (27% vs. 22%, 52% vs. 44%, and 63% vs. 52%, respectively). DEB-TACE was associated with better tolerability and a significantly lower incidence of side effects (p = 0.0001).	N/a	N/a	Some concerns
14.	Lee et al. (36) (2016)	N/a	DEB-TACE= 106 cTACE= 144	Retrospective cohort study	Doxorubicin 70mg DC Bead (Biocompatibles, UK)	Doxorubicin +Odised oil (lipiodol; Guerbet, Aulnay-sous-Bois, France) +gelatin sponge particles (Gelfoam; Upjohn, Kalama-zoo, MI)	The median PFS in the cTACE group was longer than in the DEB-TACE group (13.3 versus 10.8 months; p=0.023). There were no significant differences for OS in the DEB-TACE and cTACE groups (46.6 vs. 44.9 months; p=0.660)	**/**/**	N/a	
15.	Liang B. et al. (37) (2020)	11.0	DEB-TACE=171 cTACE=164	Retrospective cohort study	80 mg of epirubicin CalliSpheres (Jiangsu Hengrui Medicine Co., Ltd., Jiangsu Province, People’s Republic of China) 100–300 μm or 300–500 μm	Epirubicin 50–80 mg or epirubicin 50–80 mg, cisplatin, oxaliplatin or lobaplatin 50–100 mg, and 5-Fu or floxuridine 1.0 g + ethiodized oil	Post-treatment CRs were significantly higher in CSM-TACE compared to the cTACE group. PFS (25.3 months vs. 24.2 months, P=0.503) and OS (27.8 months vs. 25.3 months, P=0.203) were identical between the two groups.	**/**/***	N/a	
16.	Liu YS. et al. (38) (2015)	8	DEB-TACE=53 cTACE=64	Retrospective Case series	70 mg of doxorubicin (DC Beads; Biocompatibles, Farnham, United Kingdom) 300–500 μm	50 mg of doxorubicin + lipiodol + 500–700 μm gelatin sponges (Spongostan standard, Johnson & Johnson, Gargrave, Skipton, United Kingdom)	In the DEB-TACE group (p <0.001), more patients achieved CR compared to cTACE (32.1% vs. 6.3%). Fewer patients (p <0.001) in the DEB-TACE group had PD compared to the cTACE group (34.0% vs. 57.8%). The complication rate was higher in the cTACE group compared to DEB-TACE (54.7% and 5.7%, respectively).	N/a	Good	
17.	Liu YS. et al. (39) (2018)	60	DEB-TACE=72 cTACE=201	Retrospective Case series	Doxorubicin 70 mg (DC Bead, Biocompatibles, Farnham, United Kingdom) 300 to 500 μm	Doxorubicin 50 mg +lipiodol +500 to 700 μm gelatin sponge (Spongostan standard, Johnson & Johnson, Gargrave, Skipton, United Kingdom) or 100 to 300 μm Embosphere microspheres	The PFS was 11.0 months for cTACE and 16.0 months for DEB-TACE (P = 0.019). OS was 37 months in both treatment groups.	N/a	Good	
18.	Ma Y. et al. (40) (2019)	11.4	DEB-TACE=94 cTACE=98	Retrospective cohort study	Pirarubicin 60 mg or 80 mg CalliSpheres (Jiangsu Hengrui Medicine Co., Ltd., Jiangsu Province,China) 100-300 μm or 300-500 μm	Pirarubicin 60 mg or 80 mg +ethiodized poppyseed oil (EPO) (Jiangsu Hengrui Medicine Co., Ltd., Jiangsu Province, China) + Polyvinyl Alcohol (PVA) particles (CookMedical LLC, Bloomington, USA)	CR) and ORR were higher in the DEB-TACE group, while the DCR level was similar compared to the cTACE group. There were no differences in PFS or OS between the DEB-TACE and cTACE groups. DEB-TACE showed a higher incidence of pain and fever during treatment or hospitalization.	**/**/***	N/a	
19.	Malagari K. et al. (41) (2010)	12	DEB-TACE=41 cTACE=43	Prospective Randomized study	Doxorubicin 150 mg (DEB-TACE; DC Beads; Biocompatibles, Terumo) 100–300 and 300–500 lm,	Doxorubicin 150 mg	CR was observed in 11 patients (26.8%) in the DEB-TACE group and in 6 patients (14%) in cTACE. PR in 19 patients (46.3%) with DEB-TACE and 18 (41.9%) with cTACE. PFS was higher in DEB-TACE (42.4 ± 9.5 and 36.2 ± 9.0 weeks) (p = 0.008).	N/a	N/a	
20.	Massani M. et al. (42) (2017)	12	DEB-TACE=28 cTACE=54	Retrospective cohort study	Doxorubicin 50 mg (DCBEADS, Biocom- patibles; UK) 100–300 lm	Farmorubicin 50 mg +odized oil (Lipiodol UltraFluid; Ethiodol USA) + gelatin sponge particles	In the DEB-TACE group, OS was 22.7 months (CI 11.6–33.8), in cTACE it was 21.8 months (CI 15.7–27.9).	***/*/***	N/a	
21.	Rahman et al. (43) (2016)	11.8	DEB-TACE=45 cTACE=34	Retrospective cohort study	50-75 mg of doxorubicin	5-50 mg of doxorubicin	OS in the c-TACE and DEB-TACE groups was 4.9 ± 3.2 months and 8.3 ± 2.0 months, respectively (p=0.008). There was no statistically significant difference between the two groups regarding the mRECIST criteria.	**/**/**	N/a	
22.	Shi Q. et al. (44) (2020)	14.3	DEB-TACE=46 cTACE=52	Retrospective Case series	80 mg epirubicin CalliSpheres (Jiangsu Hengrui Medicine Co. Ltd., Jiangsu, China) 100-300 μm or 300-500 μm	10–30 mg epirubicin +lipiodol +gelatin sponge particles (300–500 μm or 500–700 μm; Alicon medical Co., Hangzhou, China)	The PFS of the DEB-TACE group and the C-TACE group was 12.0 months and 7.0 months (P < 0.001), and the OS was 21.0 months and 14.0 months (P = 0.035), respectively. DEB-TACE had a better ORR (76.1% vs. 40.4%, P < 0.001) and DCR (91.3% vs. 75.0%, P = 0.033) compared to the C-TACE group. The complication rate was identical between the two groups (67.3% vs. 57.7%, P = 0.323).	N/a	Good	
23.	Shimose S. et al. (45) (2020)	N/a	DEB-TACE=76 cTACE=98	Retrospective cohort study	30 mg of epirubicin DC-beads (Eisai Co. Ltd., Tokyo Japan) 100–300 μm	30 mg epirubicin +Lipiodol (Guerbet, Tokyo, Japan) +gelatin sponge particles (Nihon Kayaku, Tokyo, Ja- pan).	The PFS in the C-TACE and DEB-TACE groups were 8.1 and 6.1 months, respectively (p = 0.79). OR and DCR scores were 64 and 71% in patients with C-TACE and 69 and 78% in patients with DEB-TACE, respectively (p = 0.25). Complications were more common after C-TACE than DEB-TACE (p <0.001).	**/**/**	N/a	
24.	Song et al. (46) (2012)	18	DEB-TACE=60 cTACE=69	Retrospective cohort study	50 mg of doxorubicin. DC bead (DC bead, Biocompatibles, Surrey, UK) 100–500 lm	doxorubicin (40– 60 mg) or epirubicin (40–60 mg) and cisplatin (60–70 mg) +lipiodol +gel- foam or PVA particles	The radiological response was higher in the DEB-TACE group than in cTACE (p <0.001). PFS was significantly better in the DEB-TACE group than in cTACE (11.7 and 7.6 months, respectively, p = 0.018).	**/**/***	N/a	
25.	Tang J. et al. (20) (2022)	14.0	DEB-TACE=64 cTACE=70	Retrospective Case series	Pirarubicin (60mg) DC bead CalliSpheres HepaSpheres	Pirarubicin (40-50mg) + lipiodol +gelatin sponge particles	ORR was higher in the DEB-TACE group (71.9% vs. 47.3%, P = 0.008). PFS (11.5 months vs. 6.5 months P = 0.014) and OS) (18.5 months versus 13.0 months, P = 0.025) were longer in the DEB-TACE group compared to the cTACE group.	N/a	Good	
26.	Wen P. et al. (47) (2019)	18.5	DEB-TACE=52 cTACE=68	Prospective Cohort Study	Callispheres (Jiangsu Hengrui Medicine Co, Ltd., Jiangsu, P.R. China) 100 μm and 300 μm HepaSphere (Merit Medical, South Jordan, UT, USA) 50–100 μm	Adriamycin solution +lipiodol	DEB-TACE achieved a higher CR (30.8%) compared to cTACE (7.4%). In the DEB-TACE group, the median PFS was 15 months (95% CI:12-18 months) in cTACE, the median PFS = 11 months (95% CI: 10-12). The median OS is greater in DEB-TACE for 25 months versus 21 months in cTACE.	N/a	Good	
27.	Wiggermann P. et al. (23) (2011)	8.1	DEB-TACE=22 cTACE=22	Retrospective Case series	50 mg epirubicin DC Beads (Contour SE; Boston Scientific) 300–500 µm	20 mg cisplatin +lipiodol +particle embolization (Contour SE; Boston Scientific)	OR and SD for DEB- TACE were 22.7% and 68.2%. For cTACE OR 22.7 and SD 31.8%. After DEB-TACE, OS increased significantly from 651 ± 76 days versus 414 ± 43 days for cTACE (p=0.01).	N/a	Fair	
28.	Wu B. et al. (48) (2018)	6.0	DEB-TACE=24 cTACE=30	Retrospective Case series	Doxorubicin 60–80 mg/20 ml CalliSpheres Beads (Jiangsu Hengrui Medicine Co. Ltd., Jiangsu, China) 300–500 μm or 100–300 μm	Doxorubicin 10– 20 mg +lipiodol +gelatin sponge	The radiological response in the DEB-TACE group was significantly higher than in the cTACE group (p <0.05). Relapses within 6 months were more frequent in cTACE compared to DEB-TACE (43.3 vs. 16.7%; p = 0.036). The incidence of complications in the DEB-TACE group was lower (p < 0.05).	N/a	Good	
29.	Xiang H. et al. (49) (2019)	12.7	DEB-TACE=36 cTACE=37	Retrospective cohort study	pirarubicin 60 or 80 mg CalliSpheres (Jiangsu Hengrui Medicine Co, Ltd, Jiangsu Province, China) 100–300 μm or 300-500 μm	pirarubicin of 60 mg or 80 mg +lipiodol +polyvinyl alcohol (PVA) particles (Cook Medical LLC, Bloo- mington)	PFS was better in the DEB-TACE group. The frequency of adverse events between the 2 groups was the same. DEB-TACE showed a better response to treatment and progression-free survival with equal safety compared to cTACE.	**/**/***	N/a	
30.	Xiao Y. et al. (50) (2019)	N/a	DEB-TACE=26 cTACE=32	Retrospective Case series	80 mg of pirarubicin Callispheres (Jiangsu Hengrui Medicine Co, Ltd., Jiangsu, P.R. China) 100–700 μm	40 mg pirarubicin + iodized oil (Lipiodol, Guerbet Group) polyvinyl alcohol (PVA)	The ORR level in the DEB-TACE group is better than in the cTACE group. PFS was 346 and 274 days in the DEB-TACE group and cTACE group, respectively. There was no significant difference in survival rates between the two groups (P=0.081).	N/a	Fair	
31.	Zhang Z. et al. (51) (2019)	N/a	DEB-TACE=56 cTACE=33	Retrospective Cohort Study	Epirucibin 100 mg DC beads (Biocompatibles, Farnham, United Kingdom) 100-300 or 300-500 μm. Callispheres beads (Jiangsu Hengrui Medicine Co., Ltd., Jiangsu, China) 100-300 or 300-500 μm.	Doxocubicin (20-40 mg/m2) and oxaliplatin (85 mg/m2) +lipiodol (Guerbet, France) +gelfoam particles (Hangzhou Aili Kang Pharmaceutical Technology Co. Ltd., China) +polyvinyl alcohol particles (Hangzhou Aili Kang Pharmaceutical Technology Co. Ltd., China) or Embospheres (Merit Medical, South Jordan, UT, USA)	The DCR level did not differ between cTACE and DEB-TACE (p>.05), although the total DCR was higher in cTACE than DEB-TACE (1 month: 87.5% vs. 80.0%, p=.001; 3 months: 78.5% vs. 72.1%, p=.02).	**/*/**	N/a	
32.	Zhao G. et al. (52) (2021)	9.9.	DEB-TACE=42 cTACE=47	Retrospective Cohort Study	CalliSpheres (Jiangsu Hengrui Medicine Co., Ltd., Jiangsu Province, China) 100–300 μm	N/a	The CR and ORR in the DEB-TACE group were better compared to the cTACE group. There was no difference in PFS and OS between the two groups. Pain syndrome was more common in the DEB-TACE group than in the cTACE group.	**/*/***	N/a	

Stars awarded for each quality item serve as a quick visual assessment: Selection (Maximum 3 stars)/Comparability (Maximum 2 stars)/Outcome (Maximum 3 stars); N/a = not available.

### 3.2 Initial characteristics and quality assessment

32 studies were included in this meta-analysis. These studies were published between 2010 and 2024. We have discovered and added 11 new studies. 3 scales were used to assess the methodological quality of articles: Newcastle-Ottawa, NIH quality assessment tool for case series studies and Cochrane Risk of Bias (ROB) 2.0 tool. The presented research quality was predominantly low and average (**Table 2**).

### 3.3 Clinical trial

#### 3.3.1 Evaluation of the effectiveness of the procedure according to the mRECIST criteria

The effectiveness was assessed according to the mRECIST criteria: Complete Response (CR), Partial Response (PR), Stable Disease (SD), and Progressive Disease (PD). And was analyzed in two groups (455 patients with DEB-TACE and 502 cTACE patients).

**Figure 2:**
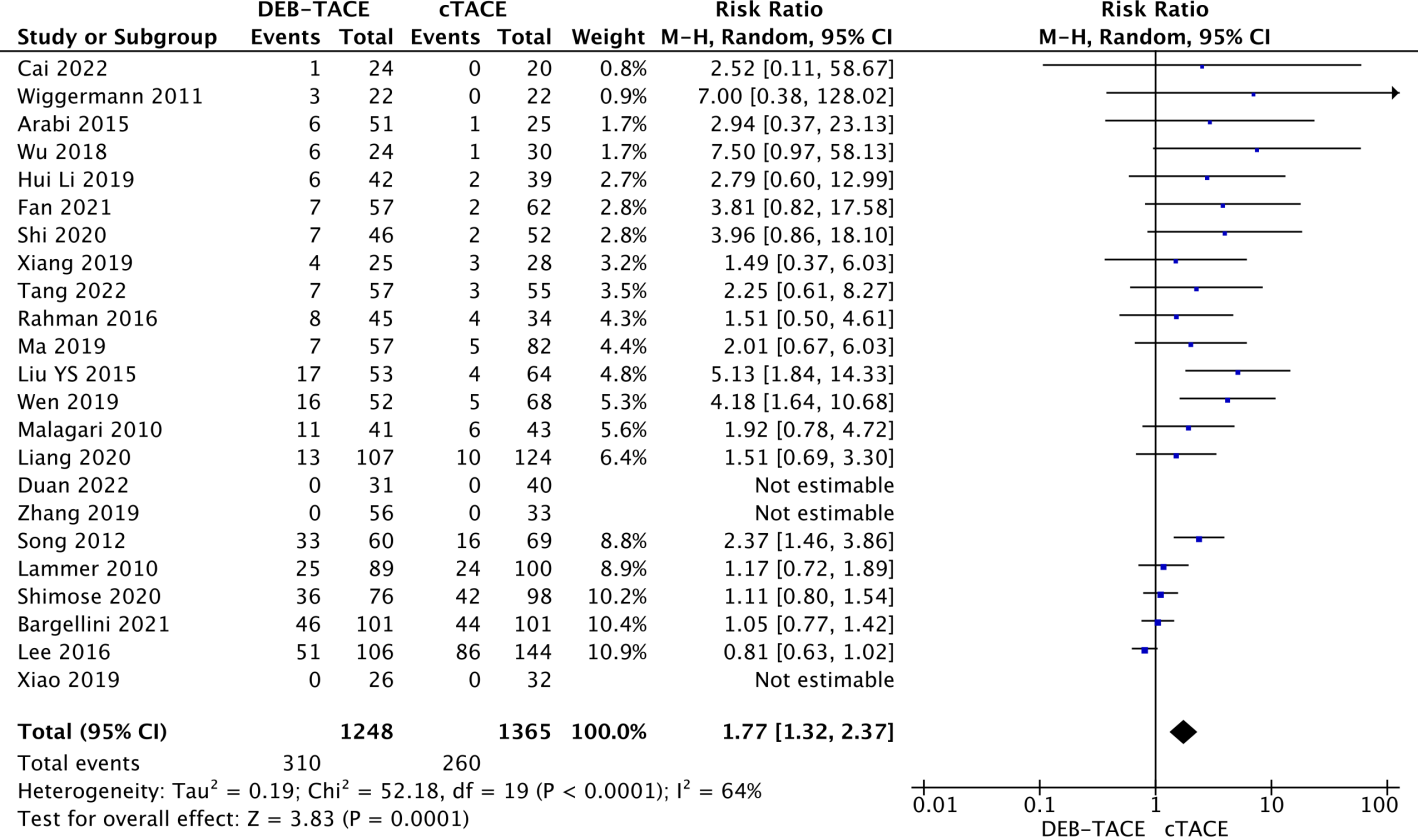
Forest plot of the complete response rate (CR) according to the mRECIST criteria.

The complete response in the DEB-TACE group was obtained in most cases compared to cTACE (310/1248) versus (260/1365) (RR, 1.77; 95% CI, 1.32 to 2.37; p=0.0001; I2 = 64%; random effects model (**Figure 2**).

**Figure 3:**
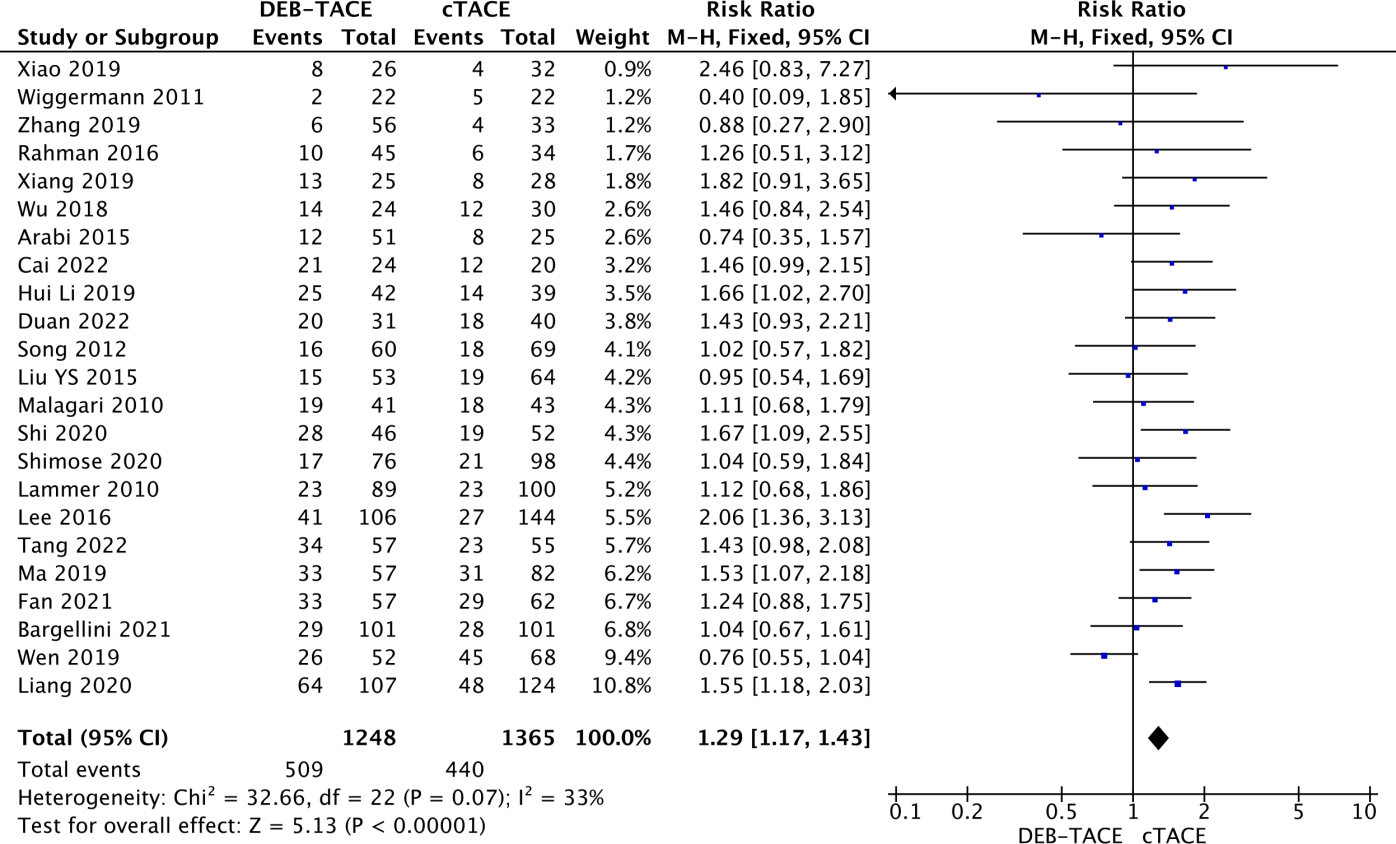
Forest plot of partial response rate (PR) according to mRECIST criteria.

A partial response was also more often recorded in the DEB-TACE group (509/1248) versus (440/1365) (RR, 1.29; 95% CI, 1.17 to 1.43; p <0.00001; I2 = 33%; fixed effects model) (**Figure 3**).

**Figure 4:**
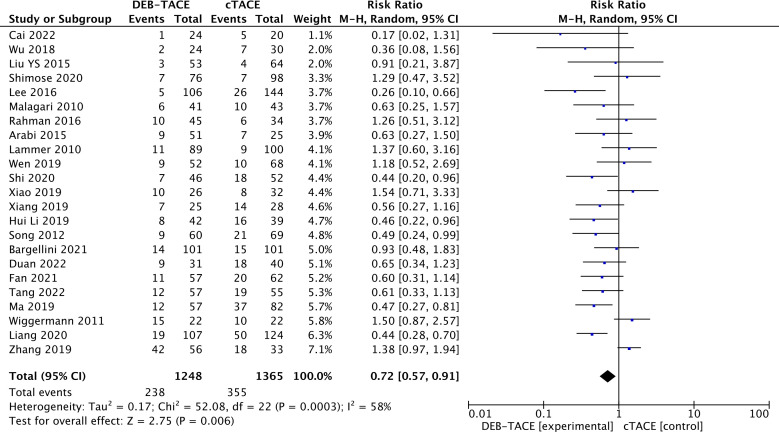
Forest plot of the frequency of Stable Disease (SD) according to mRECIST criteria.

Stabilization of the disease prevailed in the cTACE group (238/1248) than in the DEB-TACE 355/1365 group (RR, 0.72; 95% CI, 0.57 to 0.91; p=0.006; I2 = 58%; random effects model) (**Figure 4**).

**Figure 5:**
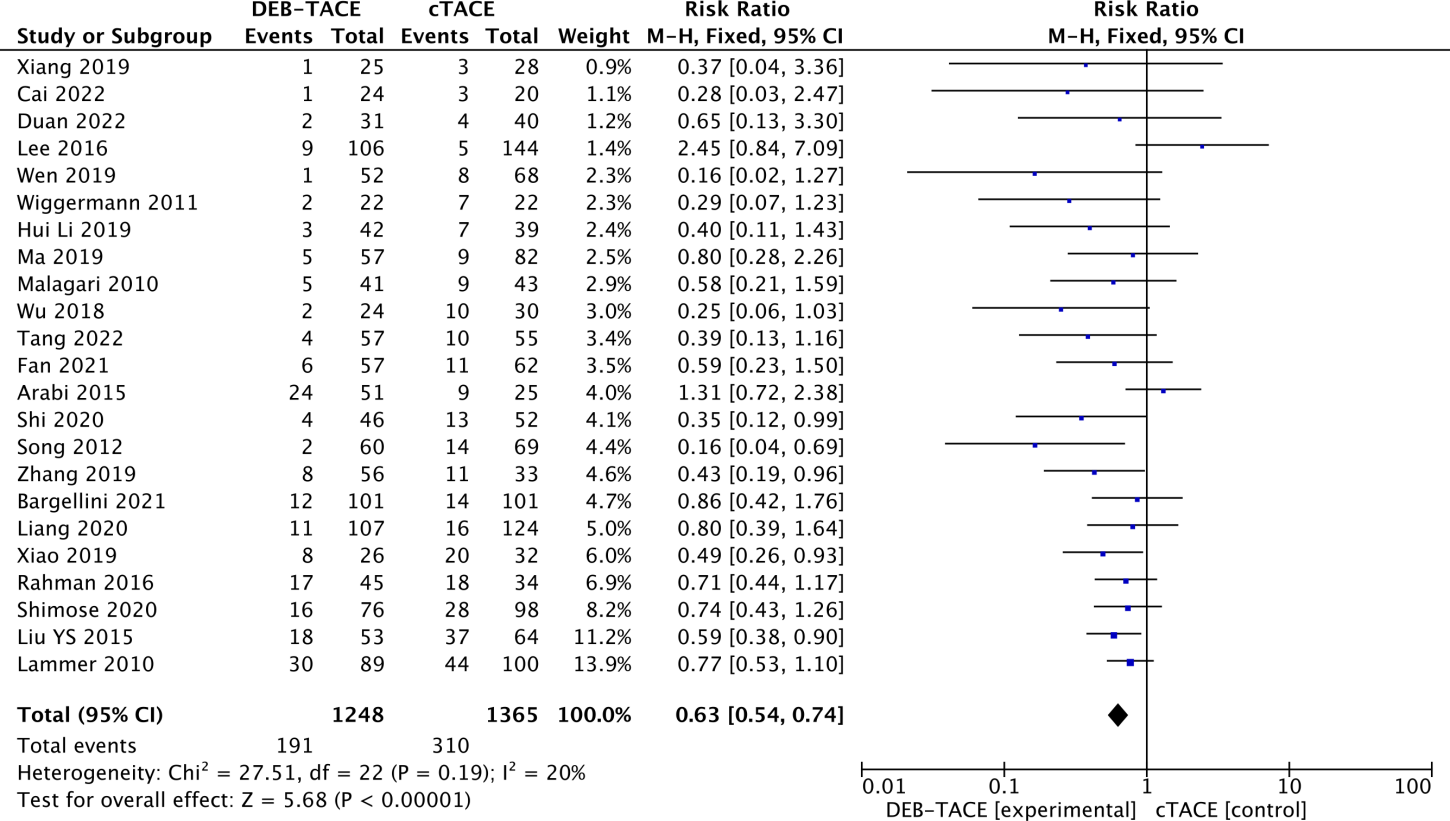
Forest plot of the frequency of Progressive Disease (PD) according to the mRECIST criteria.

Disease progression was 310/1365 (22.7%) in the cTACE group and 191/1248 (15.3%) in the DEB-TACE group (RR, 0.63; 95% CI, 0.54 to 0.74 p <0.00001; I2 = 20%; fixed effects model) (**Figure 5**).

#### 3.3.2 Overall survival rate

**Figure 6:**
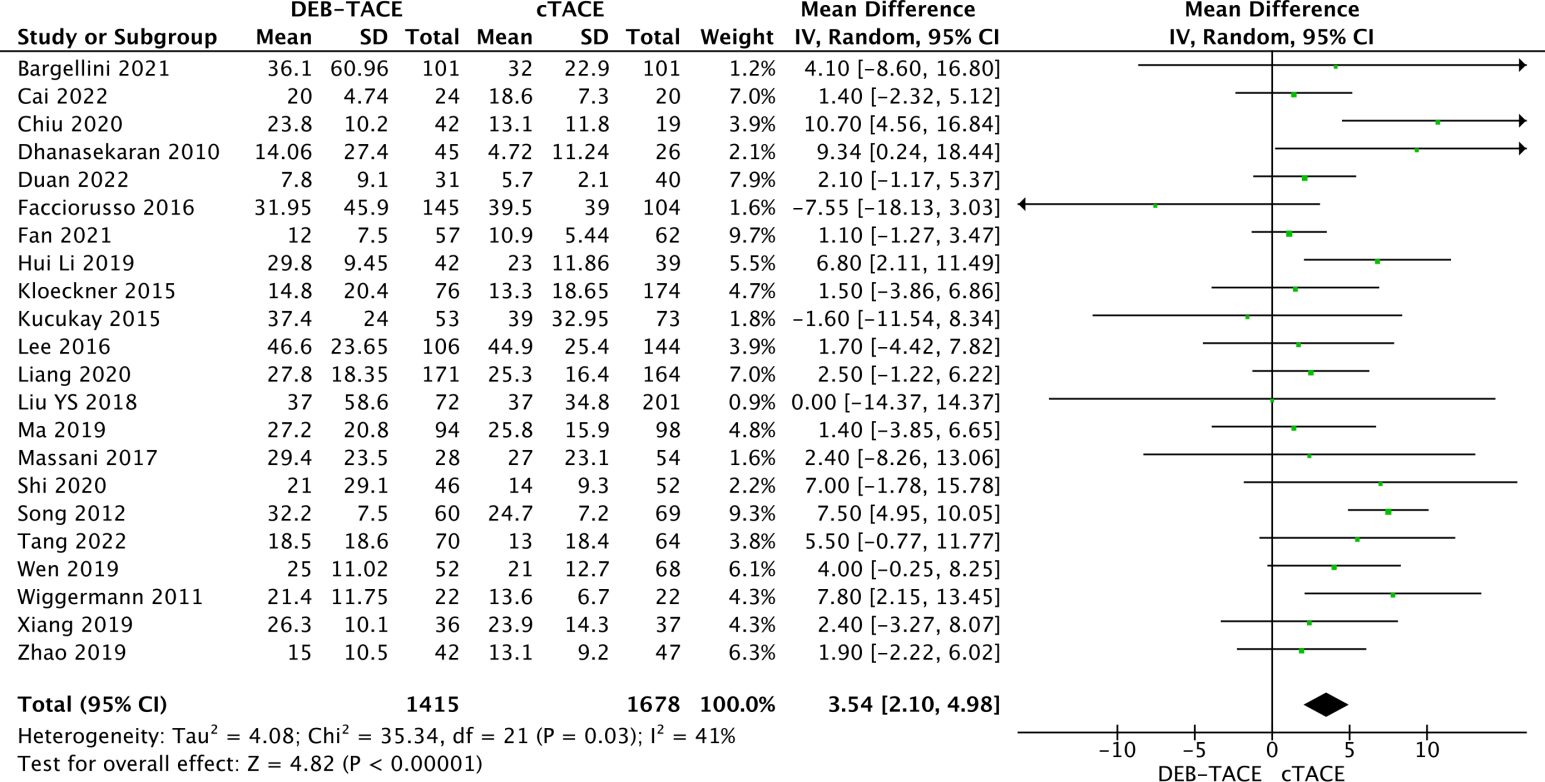
Forest plot of overall survival.

Information on overall survival is presented in 22 studies. The analysis obtained a statistically significant result in the form of better overall survival in the DEB-TACE group over cTACE (MD, 3.54; 95% CI, 2.10 to 4.98; p <0.00001; I2 = 41%; random effects model) (**Figure 6**).

#### 3.3.3 Progression-free survival

**Figure 7:**
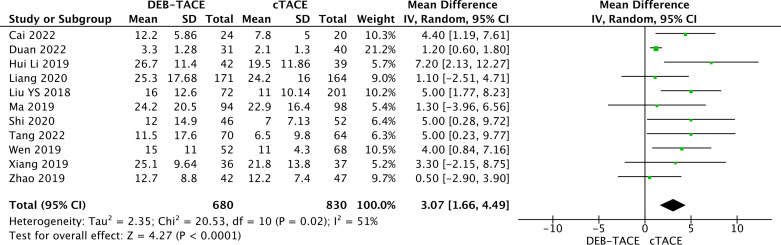
Forest plot of progression-free survival.

The analysis obtained a statistically significant result in the form of better progression-free survival in the DEB-TACE group over cTACE (MD, 3.07; 95% CI, 1.66 to 4.49; p <0.0001; I2 = 51%; random effects model) (**Figure 7**).

#### 3.3.4 Complications

**Figure 8:**
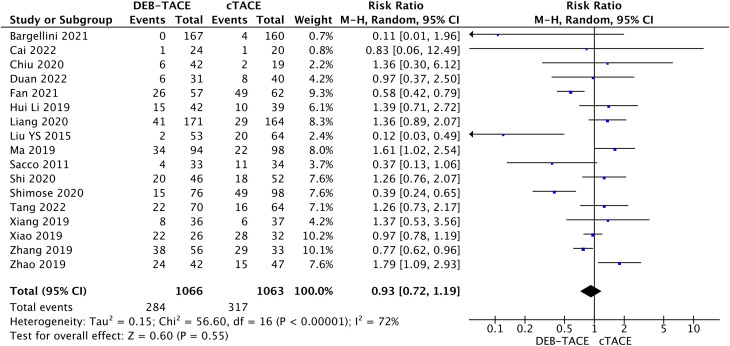
Forest plot of complications identified after hospitalization.

17 studies reported complications after treatment 284/1122(25.31%) in the DEB-TACE group and 317/1117 (28.38%) in the cTACE group (RR, 0.93; 95% CI, 0.72 to 1.19; p=0.55; I2 = 72%; random effects model (**Figure 8**).

### 3.4 Evaluation of the publication bias

**Figure 9:**
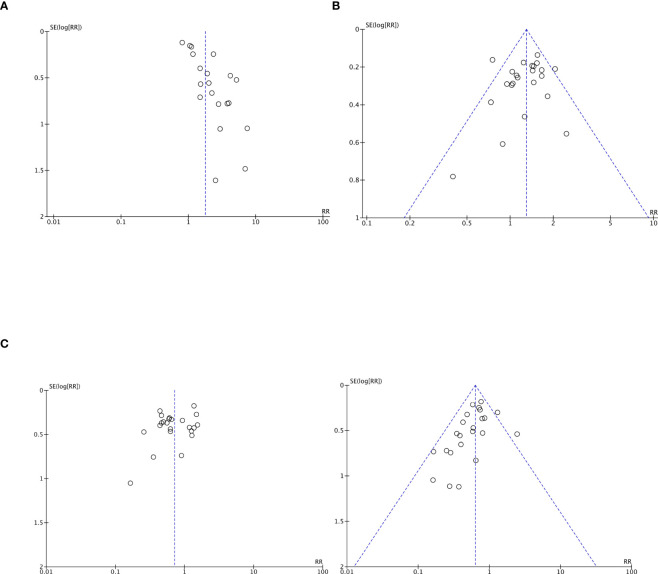
Funnel-shaped diagrams of tumor efficacy. **(A)** Complete Response. **(B)** Partial Response. **(C)** Stable Disease. **(D)** Progressive Disease .

**Figure 10:**
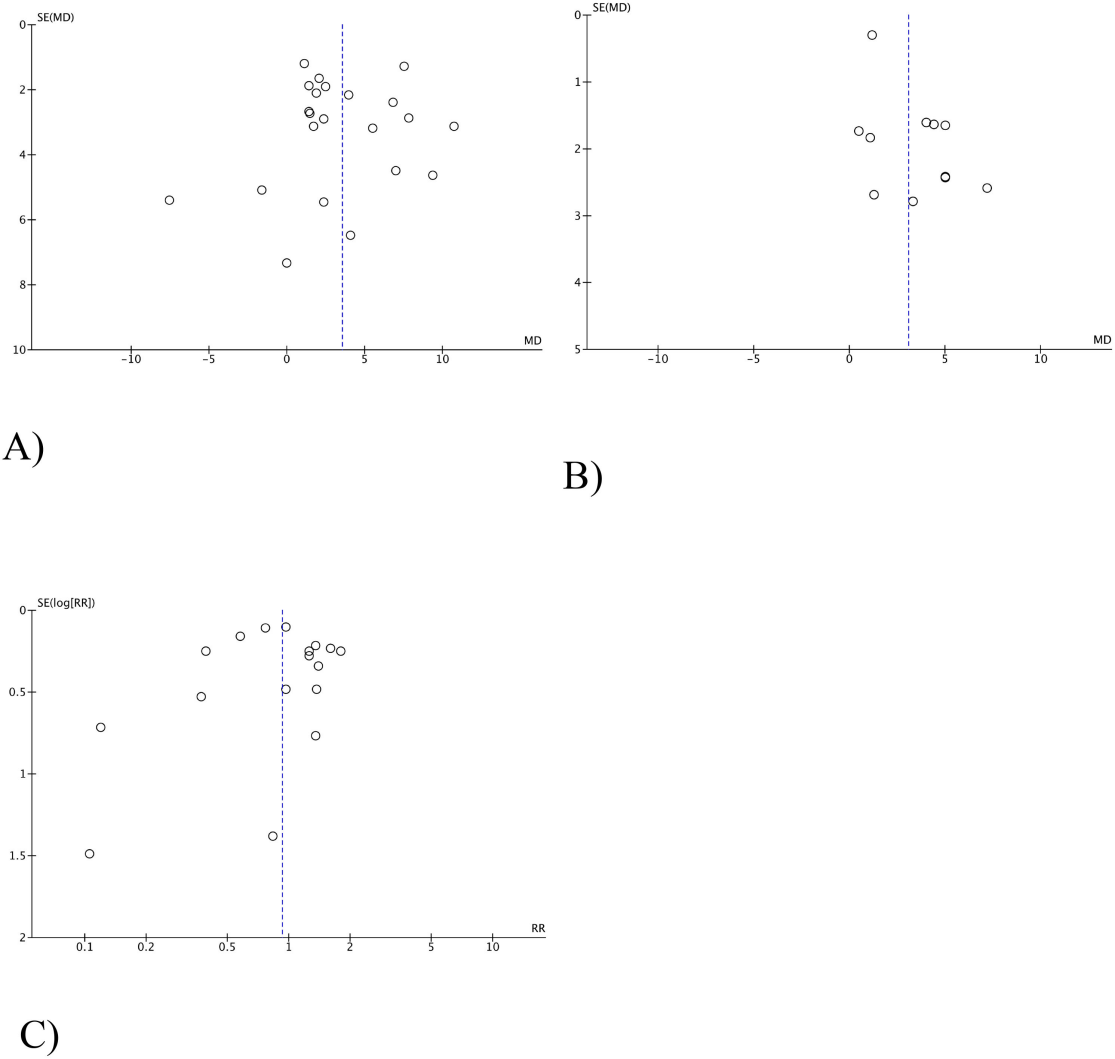
Funnel-shaped diagrams of OS **(A)**, PFS **(B)**, complications after treatment **(C)**.

The estimation of the publication bias for each research parameter was performed using a visual analysis of the funnel diagram. The studies were almost symmetrically distributed on both sides of the vertical line, which indicates a relatively small distortion of publications (**Figures 9**, **10**).

## 4 Discussion

In recent years, indications for the TACE procedure have expanded. Starting from treatment as a first-line for the intermediate stage of HCC and ending with palliative care for late-stage patients (14). Various embolic agents for transarterial embolization have been developed, the improvement of the properties of which improved clinical results (7) and dictated the need to study the dependence of the drug delivery method and its effectiveness. Previous meta-analyses (15–18) did not demonstrate definitive conclusions and led to the continuation of the publication of comparative clinical studies (19–21). Our meta-analysis is a summary of the intermediate outcome of these efforts.

According to the results of our study, it was revealed that patients in the DEB-TACE group had a clinically and statistically significantly better radiological tumor response according to the mRECIST criteria compared with cTACE. The overall survival and progression-free survival rates were significantly higher in the DEB-TACE group. At the same time, DEB-TACE did not have an increased complication rate compared to cTACE. The results obtained in the DEB-TACE group may influence the selection of patients for surgical resection, transplantation and chemotherapy line.

Previous meta-analyses comparing treatment responses between DEB-TACE and cTACE in HCC have yielded contradictory results (15–18), which is probably caused by differences between the included studies and population heterogeneity. The initial meta-analysis by Wang et al. (2020) (16) did not reveal any differences in overall survival, radiological response, and complication rates in the cTACE and DEB-TACE groups. Subsequently, Bzeizi et al. (2021) (17) evaluated the safety profile and found that DEB-TACE is associated with a better objective response (CR+PR) (OR: 1.33, 95% CI: 0.99–1.79, p<0.01), lower mortality (OR: 0.32, 95% CI: 0.16-1.17, p=0.04), fewer side effects (OR: 0.74, 95% CI: 0.24-2.24, p<0.01). However, the safety results were based on very limited data. In a meta-analysis by Wang et al. (2023) (15), the best tumor response (OR) was obtained in the DEB-TACE group (RR: 1.27, 95% CI: 1.08–1.48; p = 0.003). The overall survival time was slightly longer in the DEB-TACE group (RR: 1.05, 95% CI: 0.99–1.11, p=0.08), but the result was not statistically significant. The incidence of adverse events was slightly higher in the cTACE group (RR: 1.11, 95% CI: 0.99–1.26; p=0.08). Liang et al. (2021) (18) showed that patients who underwent DEB-TACE had the best complete response (CR) (OR: 2.00, 95% CI: 1.29–3.09, p=0.89), objective response (ORR) (OR: 2.87, 95% CI: 2.15–3.83, p=0,96). Four studies presented PFS and OS data and were included in the combined analysis. The combined results showed a tendency towards longer duration of PFS (HR: 0.86, 95% CI: 0.67–1.11, p=0.16) and OS (HR: 0.79, 95% CI: 0.59–1.07, p=0.58) with DEB-TACE compared to cTACE, although these differences did not reach statistical significance. The analysis of the safety profile revealed no differences in the frequency of adverse events.

Previous studies have not shown that DEB-TACE demonstrates a significant improvement in overall survival or tumor response rate compared to cTACE, calling into question the broader clinical benefits of this technique despite targeted drug delivery. However, the presence of a statistically significant advantage of DEB-TACE in overall survival and tumor response rate in some studies gave impetus to further research in this area, which led to the need to conduct an updated meta-analysis. Our work is the result of efforts and summarizing the results of previous research. The results obtained are statistically and clinically significant. The radiological response of the tumor in all four parameters CR, PR, SD, PD in the DEB-TACE group showed the best response (RR, 1.77; 95% CI, 1.32 to 2.37; p =0.0001; I2 = 64%; RR, 1.29; 95% CI, 1.17 to 1.43; p <0.00001; I2 = 33%; RR, 0.72; 95% CI, 0.57 to 0.91; p =0.006; I2 = 58%; RR, 0.63; 95% CI, 0.54 to 0.74 p <0.00001; I2 = 20%; respectively). The overall survival rate during the DEB-TACE procedure was higher by 3.54 months (p <0.00001), and progression-free survival (PFS) by 3.07 months (p <0.0001), respectively. At the same time, the incidence of complications was comparable in both groups. Although, in some cases DEB-TACE can cause more serious side effects such as bile duct damage (60, 61). Controlled, sustained drug release can lead to prolonged local toxicity, which should be considered when administering DEB-TACE (62).

The results obtained during the meta-analysis can significantly affect the practice of using TACE. Thus, when using TACE as a Bridge therapy, in order to reduce tumor progression and the frequency of patients dropping out of the waiting list for liver transplantation, the overall survival of the patient is crucial. Choosing DEB-TACE technology can clinically significantly increase the survival time and increase the chances of liver transplantation. The best radiological response in the DEB-TACE group can be used in down-standing therapy to lower the tumor stage, which can increase the patient’s chances of resection surgery. The radiological response and increased survival time in the DEB-TACE group can significantly affect the use of antitumor drug therapy, changing the choice of therapy line, the algorithm of further management and the timing of follow-up. And also better integrate the use of image segmentation with deep learning technologies in the evaluation of treatment results (58, 59).

There are a number of fundamental limitations in our work. Most of the studies were not randomized and were retrospective in nature, which can lead to a variety of systematic biases, including selection bias, attrition bias, reporting bias and other systematic and random errors. In the included trials, patients were selected according to the BCLC classification with stages A and B. Some studies included only patients in stage B, while others included both B and A. These selection criteria may influence the heterogeneity of the patient groups, which may affect prognosis and overall survival rates. Many aspects of the technical implementation of both types of chemoembolization were not taken into account in the meta-analysis process. The type of embolizing agent material leads to a different ability to adsorb the chemotherapy drug and retain it for a long time in the bloodstream during embolization, which affects the local concentration of the chemotherapy drug and systemic toxicity. In addition, DEB-TACE may require more precise planning and monitoring because of the sustained release mechanism of the beads and the possibility of embolization complications. Furthermore, the size of the emboli reflects the selectivity of delivery of the chemotherapy drug to the tumor, determining the degree of ischemia of healthy tissue. While DEB-TACE offers the advantage of customizable bead sizes, selecting the wrong size can lead to suboptimal outcomes, including inadequate embolization or excessive tissue ischemia (53, 54). However, given the different size of the emboli used, we did not consider this factor in our analysis. Further studies are needed to assess the risks of non-targeted obstruction (55). In addition, in some clinical cases, a differentiated approach to transarterial chemoembolization techniques is required. For example, DEB-TACE releases chemotherapeutic agents in a controlled manner, but this may limit the extent of drug distribution compared to the oil-based emulsions used in cTACE. It may also affect treatment efficacy in larger or more vascularized tumors (56, 57). The chemotherapy drug group also affects the level of response to HCC. Systemic administration of different groups of drugs causes a heterogeneous tumor response. Local administration of the same drugs can similarly lead to different changes in tumor cells, which can affect the overall survival and radiological response (22, 23). These features were not taken into account during the meta-analysis, and there was significant heterogeneity in the presented works with respect to the emboli and chemotherapeutic drugs used. In addition, DEB-TACE uses drug-eluting beads, which are more expensive than the materials used in cTACE. This may make it less affordable in resource-limited settings. This should be taken into account when comparing treatment effects and planning oncology programs. Another limitation of our research was the analysis of publications in English only.

## 5 Conclusion

The results of the meta-analysis revealed clinically significant advantages of DEB-TACE in comparison with cTACE. Being comparable in the frequency of complications, DEB-TACE demonstrated the best results in the radiological response of the tumor to the therapy, in terms of overall survival and progression-free survival, which may affect the selection of patients for surgical treatment, as well as the choice of a line of chemotherapy. Thus, DEB-TACE may have an advantage over сTACE in increasing the overall life expectancy of patients with HCC.

The data obtained as a result of the meta-analysis are subject to distortions and systematic errors due to the small sample size, lack of randomization and the predominantly retrospective nature of the studies. To improve the methodological quality of studies, as well as an objective comparison of the effectiveness of DEB-TACE and cTACE, it is necessary to conduct prospective randomized trials on a large cohort of patients comparing the effectiveness and safety of these procedures in patients with HCC.

## Data Availability

The raw data supporting the conclusions of this article will be made available by the authors, without undue reservation.
